# Leukocyte Telomere Length in Major Depression: Correlations with Chronicity, Inflammation and Oxidative Stress - Preliminary Findings

**DOI:** 10.1371/journal.pone.0017837

**Published:** 2011-03-23

**Authors:** Owen M. Wolkowitz, Synthia H. Mellon, Elissa S. Epel, Jue Lin, Firdaus S. Dhabhar, Yali Su, Victor I. Reus, Rebecca Rosser, Heather M. Burke, Eve Kupferman, Mariana Compagnone, J. Craig Nelson, Elizabeth H. Blackburn

**Affiliations:** 1 Department of Psychiatry, University of California San Francisco (UCSF) School of Medicine, San Francisco, California, United States of America; 2 Department of OB-GYN and Reproductive Sciences, University of California San Francisco (UCSF) School of Medicine, San Francisco, California, United States of America; 3 Department of Psychiatry and Health Psychology Program, University of California San Francisco (UCSF) School of Medicine, San Francisco, California, United States of America; 4 Department of Biochemistry and Biophysics, University of California San Francisco (UCSF) School of Medicine, San Francisco, California, United States of America; 5 Department of Psychiatry and Behavioral Sciences, Stanford University School of Medicine, Stanford, California, United States of America; 6 Kronos Science Laboratory, Phoenix, Arizona, United States of America; Innsbruck Medical University, Austria

## Abstract

**Background:**

Depression is associated with an unusually high rate of aging-related illnesses and early mortality. One aspect of “accelerated aging” in depression may be shortened leukocyte telomeres. When telomeres critically shorten, as often occurs with repeated mitoses or in response to oxidation and inflammation, cells may die. Indeed, leukocyte telomere shortening predicts early mortality and medical illnesses in non-depressed populations. We sought to determine if leukocyte telomeres are shortened in Major Depressive Disorder (MDD), whether this is a function of lifetime depression exposure and whether this is related to putative mediators, oxidation and inflammation.

**Methodology:**

Leukocyte telomere length was compared between 18 unmedicated MDD subjects and 17 controls and was correlated with lifetime depression chronicity and peripheral markers of oxidation (F2-isoprostane/Vitamin C ratio) and inflammation (IL-6). Analyses were controlled for age and sex.

**Principal Findings:**

The depressed group, as a whole, did not differ from the controls in telomere length. However, telomere length was significantly inversely correlated with lifetime depression exposure, even after controlling for age (p<0.05). Average telomere length in the depressed subjects who were above the median of lifetime depression exposure (≥9.2 years' cumulative duration) was 281 base pairs shorter than that in controls (p<0.05), corresponding to approximately seven years of “accelerated cell aging.” Telomere length was inversely correlated with oxidative stress in the depressed subjects (p<0.01) and in the controls (p<0.05) and with inflammation in the depressed subjects (p<0.05).

**Conclusions:**

These preliminary data indicate that accelerated aging at the level of leukocyte telomeres is proportional to lifetime exposure to MDD. This might be related to cumulative exposure to oxidative stress and inflammation in MDD. This suggest that telomere shortening does not antedate depression and is not an intrinsic feature. Rather, telomere shortening may progress in proportion to lifetime depression exposure.

## Introduction

Major depressive disorder (MDD) is associated with a significantly increased risk of developing serious medical illnesses that are more commonly seen with advanced age, such as diabetes, cardiovascular disease, immune impairments, stroke, dementia, osteoporosis, diabetes and metabolic syndrome [1], [2], [3], [4], [5] and of dying significantly earlier (even after accounting for socio-demographic factors, suicide and risk factors such as smoking, alcohol and physical illness) [6], [7], [8], [9], [10]. Indeed, major depression has been likened to a state of “accelerated aging,” with an increased incidence of aging-related illnesses [1], [8], [11], [12], [13], [14], [15], [16], [17], [18], [19], [20], [21], [22]. Various explanations for a prematurely aged phenotype in depression have been proposed, such as the “glucocorticoid cascade” hypothesis [23], [24] and “allostatic load” [25], as well as an unhealthy lifestyle or environment. In this study, we explored the additional (and not mutually exclusive) possibility that “accelerated aging” in depression occurs at the level of telomeres, as manifest in blood leukocytes. Further, we hypothesized that such changes are directly correlated with cumulative lifetime exposure to depression and are related to specific cytotoxic biochemical mediators, such as pro-inflammatory cytokines and oxidative stressors, which are often elevated in depression.

Telomeres are deoxyribonucleic acid (DNA)-protein complexes that cap the ends of the linear chromosomal DNA, protecting the genome from damage. In mitotic cells, telomeres can shorten with each division, unless this can be counteracted or reversed by the telomere-lengthening enzyme, telomerase [26], [27]. When telomeric DNA reaches a critically short length, as in cells undergoing repeated mitotic divisions (e.g., leukocytes [28] and stem cells [29], [30], [31], [32], [33]), cells become susceptible to senescence or apoptosis [26], [34]. Even in non-dividing cells, telomere shortening has been associated with cytotoxic stressors such as oxidative stress, which preferentially damages telomeric DNA compared with non-telomeric DNA, and chronic inflammation [35], [36], [37], [38]. Such enhanced telomere shortening also increases cellular susceptibly to apoptosis and death [33]. Telomere length is emerging as a prognostic marker of disease risk and a robust indicator of human “biological age” (as distinguished from chronological age). It may represent a cumulative log of factors such as the number of cell divisions and of exposure to cytotoxic processes such as excessive oxidation and inflammation [34], [35], [38], [39], [40], [41], [42]. Several recent studies in non-depressed populations have demonstrated an inverse relationship between leukocyte telomere length and the risk of current and future medical illnesses (such as cardiovascular and infectious diseases and dementia) and early death [42], [43], [44], [45], [46], [47], [48], [49], [50], [51], [52], [53], [54]. Accelerated telomere shortening in individuals with MDD could help explain the increased medical morbidity seen in depression [8], [40], [46], [55], [56], [57].

In addition to biochemical stressors such as oxidation and inflammation, chronic psychological stress is also associated with shortened telomeres [58], [59]. For example, healthy chronically stressed individuals (e.g., maternal caregivers of chronically ill children and family caregivers of demented individuals) showed significantly shorter leukocyte telomere length compared to age-matched controls [58], [59], and in one study, telomere length was inversely correlated with the chronicity of caregiving (i.e., women with greater cumulative duration of caregiving stress had shorter telomeres) [58]. This is consistent with the hypothesis that telomere shortening develops over time in proportion to cumulative exposure to the stressors. The difference in mean telomere base pairs (bp) between the two groups in that study suggested approximately 9–17 years of accelerated biological aging in the stressed, compared to the non-stressed, women [58]. In the first study of telomere length in affective illnesses, including MDD, subjects also had significantly shortened leukocyte telomere length compared to controls, with an estimated acceleration of biological cell aging of over 10 years (average telomere shortening  = 660 bp) [55]. That study utilized an extremely chronically ill population (average cumulative lifetime duration of illness =  31.8±11.2 [SD] years). To the extent telomere length reflects cumulative exposure to depression and cytotoxic factors, telomere length should be inversely correlated with the lifetime chronicity of depression, and therefore, extrapolation of findings from that study to individuals with less chronic major depression may not be possible. A second study examined leukocyte telomere shortening in major depression and also found shortened telomeres [60], but it did not report the chronicity of depression in their sample. A very recent study also found shortened leukocyte telomeres in MDD (although various psychiatric, neurological and somatic disorders were not excluded in their sample), with an average shortening of 350 bp [61], representing approximately 6–8 years of “accelerated aging.” In that study, average telomere length was not related to lifetime depression history, as assessed by the time from the first onset of MDD through the time of the present assessment, but intervening periods of time that the subjects were not depressed were not excluded in that assessment. In the present study, to test effects of depression status and chronicity on cell aging, we evaluated depressed individuals across a broad range of depression chronicity and assessed chronicity as the estimated time actually spent in depressive episodes.

In addition to investigating telomere length in depression, we were interested in assessing biological factors associated with telomere shortening. Since depression is often associated with increased oxidative stress [57], [62], [63], [64], [65], [66], [67] and with a pro-inflammatory milieu [4], [68], [69], [70], [71], [72], we reasoned that oxidative stress and inflammation might contribute to shortened telomeres in depression, just as they are believed to do in certain medical illnesses and in preclinical models [36], [38], [41], [46], [57], [73], [74], [75], [76], [77], [78]. We hypothesized that depressed individuals would have shorter leukocyte telomeres than matched controls, that telomere length would be inversely correlated with cumulative lifetime exposure to depression, and that telomere length would be inversely correlated with oxidative stress and inflammatory markers.

### Methods

### Ethics Statement

Subjects gave written informed consent to participate in this study, which was approved by the University of California, San Francisco (UCSF) Committee on Human Research (CHR).

### Objectives

To determine whether leukocyte telomeres are shortened in individuals with Major Depressive Disorder (MDD), whether this is a function of depression chronicity and whether this is related to putative mediators, oxidation and inflammation.

### Participants

Eighteen subjects with MDD, diagnosed with the Structured Clinical Interview for DSM-IV-TR (SCID) [79], and 18 individually-matched healthy controls (matched by sex, ethnicity and age ±3 years) were recruited. One healthy control had an inadequate blood sample collection, leaving 18 depressed subjects and 17 healthy controls with usable data. Depressed subjects were all outpatients; they and the controls were recruited by fliers, bulletin board notices, Craigslist postings, newspaper ads and, in the case of depressed subjects, clinical referrals. Subjects were paid for their participation. SCID diagnostic interviews were conducted by an experienced clinical psychologist and were clinically verified by a separate psychiatric interview with a Board-certified psychiatrist. Depressed subjects with psychosis or bipolar histories were excluded, although co-morbid anxiety disorders were allowed when the depressive diagnosis was considered to be the primary diagnosis. Subjects with Post-Traumatic Stress Disorder (PTSD) were excluded, since PTSD may have important differences in stress hormone regulation [80]. Seven of the depressed subjects had co-morbid anxiety diagnoses as follows: three with generalized anxiety disorder, two with obsessive-compulsive disorder, two with binge eating disorder (one of whom was in remission) and one with social anxiety disorder. Healthy controls were also screened with the SCID, and were required to have no present or past history of any DSM-IV Axis I or Axis II diagnosis. Potential subjects were excluded if they met SCID criteria for alcohol or substance abuse within 6 months of entering the study. Subjects in both groups were medically healthy (assessed by physical examination, review of systems and screening laboratory tests), had no acute illnesses or infections, and had not had any vaccinations within 6 weeks of entering the study. All subjects (depressed and control) were free of any psychotropic medications, including antidepressants, antipsychotics and mood stabilizers, as well as any hormone supplements, steroid-containing birth control or other interfering medications (e.g. statins) or Vitamin supplements above the U.S. Recommended Daily Allowances (e.g. Vitamin C, 90 mg/day), for a minimum of 6 weeks before entry into the study (with the exception of short-acting sedative-hypnotics, as needed, up to a maximum of 3 times per week, but none within one week prior to testing).

### Procedures

Subjects were admitted as outpatients to the UCSF Clinical and Translational Science Institute's Clinical Research Center at 8:00 am, having fasted (except water) since 10:00 pm the night before. Before proceeding with testing, all subjects were required to test negative on a urine toxicology screen (measuring the presence of abused drugs) and, in women of child-bearing capacity, a urine pregnancy test. After the subjects had sat quietly for 45 minutes, blood samples were obtained for leukocyte telomere length, oxidative stress markers (F2-isoprostanes and the anti-oxidant, Vitamin C) and inflammation (IL-6). Whole blood was drawn for the telomere length assay, and buffy coat was saved for leukocyte telomere length assay. DNA was prepared from whole blood using commercially available reagents (Gentra Puregene Blood Kit, Qiagen, Valencia, CA). Blood for the F2-isoprostane assay was collected into EDTA tubes with no vacuum, and blood for the Vitamin C assay was collected into foil wrapped serum separator tubes. Blood for IL-6 assay was collected into serum separator tubes.

Severity of depression in the depressed subjects was ascertained with the observer-rated 17-item Hamilton Depression Rating Scale (HDRS) [81]. Total lifetime duration of depression was estimated in the depressed subjects using the life history methods of Sheline [82] and Post [83], supplemented with information derived from the SCID interview and the Antidepressant Treatment History Form (ATHF) [84], which documents depressive episode durations as well as durations of antidepressant treatment, including the doses used and the treatment response. Only periods of time during which subjects were actively depressed (i.e., met DSM-IV criteria for MDD) were counted; periods of time during which subjects were not depressed were not included. Clinical history-taking and telomere assays were performed blind to each other.

### Assays

#### Telomere Length

High molecular weight DNA was extracted from frozen whole blood using commercially available reagents (Puregene, Gentra Systems, Qiagen, Valencia, CA). DNA quality and quantity were assessed with a nanodrop spectrophotometer and random samples were also assessed by agarose gel electrophoresis. The telomere length measurement assay was adapted from the published original method [85]. Briefly, the T (telomeric) and S (single copy gene) values of each sample were determined by quantitative polymerase chain reaction (PCR) using the following primers: *tel1b* [5′-CGGTTT(GTTTGG)5GTT-3′] and *tel2b* [5′-GGCTTG(CCTTAC)5CCT-3′] for T and *hbg1* [5′ GCTTCTGACACAACTGTGTTCACTAGC-3′] and *hbg2* [5′-CACCAACTTCATCCACGTTCACC-3′] for S (human beta-globin). Genomic DNA from HeLa cells was used as the reference to quantify the T and S values relative to the reference DNA sample by the standard curve method. All PCRs were carried out on a Roche Lightcycler 480 real-time PCR machine with 384-tube capacity (Roche Diagnostics Corporation, Indianapolis, IN). The telomere thermal cycling profile consisted of: cycling for T (telomeric) PCR: denature at 96°C for 1 second, anneal/extend at 54°C for 60 seconds, with fluorescence data collection, 30 cycles; cycling for S (single copy gene) PCR: denature at 95°C for 15 seconds, anneal at 58°C for 1 second, extend at 72°C for 20 seconds, 8 cycles; followed by denature at 96°C for 1 second, anneal at 58°C for 1 second, extend at 72°C for 20 seconds, hold at 83°C for 5 seconds with data collection, 35 cycles. The inter-assay coefficient of variation (CV) for telomere length measurement was 4%. Details of the method can be found in [86].

#### Oxidative Stress

The recommended approach for evaluating oxidative stress assesses the balance between anti-oxidants and oxidative by-products, with assessment of at least one antioxidant and one oxidized molecule [87], [88], [89]. Ascorbic acid (Vitamin C) is often the preferred antioxidant to measure in this context [87], [88], [89]. Although ascorbic acid is an essential nutrient in humans (entirely derived from dietary consumption), ascorbic acid levels reach steady state concentrations under fasting conditions (approximately six hours following food consumption), and they have been found to be relatively stable within individuals across time and to predict long-term health outcomes up to 12 years later [90], [91], [92]. Because of this, and in order to limit the problem of multiple hypothesis testing by examining oxidative by-products and anti-oxidants separately, we *a priori* defined “oxidative stress” as the ratio of F2-isoprostanes [93], [94], [95] (a class of major oxidative by-products) to ascorbic acid [89], [96]. In a prior study from our group, the ratio of oxidative by-products to anti-oxidants was found to be inversely correlated with leukocyte telomere length in stressed caregivers [58].

F2-isoprostanes (a collection of isomers) were measured by Dr. Jason Morrow's Lab at Vanderbilt University by gas chromatography-mass spectrometry (GC-MS) as described previously. [97], [98]. The isoprostanes were extracted and purified with solid phase extraction and thin layer liquid chromatography and then converted to trimethylsilyl ether derivatives and analyzed by GC-MS. Ascorbic acid was measured by Kronos Science Laboratory using high performance liquid chromatagraphy (HPLC) method as described previously [99]. Briefly, the serum sample was preserved by adding an equal volume of metaphosphoric acid and treated with dithiothreitol The resulting supernatant was injected into the HPLC system equipped with diode array detector (DAD). The separation was carried out using a 250×4.6 mm Capcell Pak NH_2_ column (Shiseido, Tokyo, Japan) with a flow rate of 1 mL/min of the mobile phases consisted of 10 mM ammonium acetate at pH 4.2 and methanol. Ascorbic acid was quantified using external standards with UV spectrophotometric detection at 243 nm wavelength.

#### IL-6

Samples were collected in 10 ml serum separator tubes (SST) tubes (Becton Dickinson, Franklin Lakes, NJ). Serum was frozen and stored at – 80 C. A high sensitivity enzyme-linked immunosorbent assay was used to quantify IL-6 concentrations (R&D Systems, Minneapolis, MN). The assay sensitivity is <0.1 pg/ml, and average intra- and inter-assay coefficients of variation are 7% and 8% respectively. Each sample was analyzed in duplicate according to manufacturer protocol. IL-6 assays were performed in the lab of Dr. Firdaus Dhabhar at Stanford University.

### Statistical Methods

We first assessed the impact of age, sex, body-mass index (BMI), and lifetime and current tobacco use as potential confounds; we found significant effects of age and sex on telomere length, and significant effects of age and BMI on IL-6 (data presented below). Lifetime and current tobacco use were not significantly related to any of these variables. Consequently, all analyses were controlled for age and sex, and analyses involving IL-6 were additionally controlled for BMI. Before analyzing the data, distributions were examined for normality; non-normal distributions were natural log transformed (Ln).

Between-group comparison of the demographic variables was by independent sample t-tests, Chi square tests and independent sample Kruskal-Wallis tests. Independent sample tests, rather than paired sample tests, were used, since the absence of blood data in one control subject (described above) resulted in unequal group sizes. Other between-group data, when covariates were applied, were analyzed by analysis of covariance (ANCOVA). Correlations between variables were assessed by linear regression, or by hierarchical linear regression, with age and sex (and BMI, in the case of IL-6) entered first. All tests were 2-tailed with an alpha =  0.05.

## Results

### Demographics

The mean age of the depressed and control subjects did not significantly differ (36.6±11.8 [SD] vs. 36.8±11.0 years [range 25–69 years], respectively), nor did the sex distribution (65% female in each group), ethnicity distribution or body-mass index (24.8±3.7 vs. 26.2±5.7 [kg/m(2)], respectively). The subject groups also did not significantly differ in current and past alcohol and nicotine consumption, marital status, highest educational level attained or self-rated socioeconomic status [100], although mean household income was significantly lower in the depressed subjects than in the controls (t =  2.59, p<0.02, df = 32). Household income was not significantly correlated with leukocyte telomere length (r =  0.13, ns, df =  25), IL-6 concentrations (r = 0.01, ns, df = 28) or the oxidative stress ratio (r =  −0.17, ns, df = 27). In addition, average activity (exercise) levels per month, as measured by the Yale Physical Activity Survey (YPAS) [101], were significantly lower in the depressed sample than in the controls (t =  2.88, p<0.01), but activity level was not significantly correlated with leukocyte telomere length (r =  0.19, ns, df), IL-6 concentrations (r = −0.01, ns, df) or the oxidative stress ratio (r =  −0.09, ns, df). The mean 17-item Hamilton Depression Rating Scale (HDRS) [81] rating in the depressed subjects was 19.3±3.9 (range 17–26), and the mean chronicity of depression (i.e., lifetime months of depression) was 156.5±134.8 months (range 9.3–425.8 months). The mean length of the current episode of depression was 125.1±158.0 months (range 1.7–425.8 months). Demographic characteristics of the subjects are provided in Table 1.

**Table 1:** Characteristics of Depressed and Control Subjects.

	Controlsn = 17	Depressedn = 18	p
Age (Years)	36.6±11.8	36.8±11.0	ns
Sex (Female, %)	65%	67%	ns
Ethnicity (%)CaucasianAfrican-AmericanAsianOther or Mixed	71%18%6%5%	72%17%6%5%	ns
Body-Mass Index (kg/m[2])	24.8±3.7	26.3±5.9	ns
No Tobacco Ever (%)	59%	67%	ns
Current Tobacco Use (%) NoneSometimesDaily	82%12%6%	83%17%0%	ns
Subjective Socio-economic Status^1^	6.45±1.13	5.75±1.60	ns
Years of Education	15.82±2.28	15.28±2.06	ns
Annual Household Income ($)	$59,775±$32,550	$29,225±$26,005	<0.02
Physical Activity Level^2^	3.11±0.90	2.10±1.26	<0.01

1 Subjective socioeconomic status was measured using a 10-rung ladder version of the MacArthur Scale of Subjective Social Status [100], with higher numbers equaling higher perceived socioeconomic status.

2 Physical Activity Level was measured with the Yale Physical Activity Survey (YPAS) [101]. On this scale, 1 =  “not very active;” 2 =  “weekend/vacations only;” 3 =  “more than 1–2 times per week;” 4 =  “more than 3 times per week.” Other measures on the YPAS, such as “Vigorous Activity” and “Duration of Vigorous Activity” yielded similar differences between groups.

### Telomere Length

**Figure 1:**
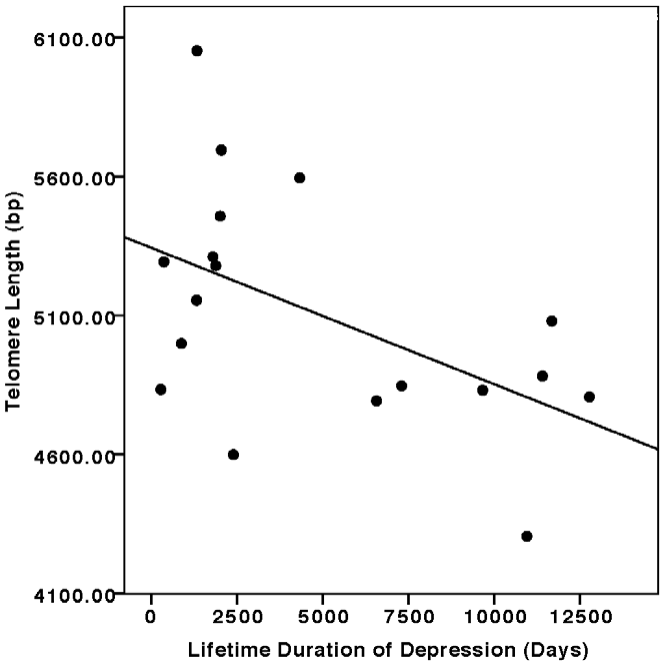
Relationship between cumulative lifetime duration of depression and leukocyte telomere length (in base pairs, bp). (F =  4.70, p<0.05, controlling for age and sex by hierarchical linear regression).

Overall, leukocyte telomere length (in bp) was not significantly different in the depressed subjects compared to the controls (mean ± SD: depressed: 5101±425 bp, vs. controls: 5141±282 bp; difference =  40 bp) (F =  0.17, ns [p = 0.66], controlling for age and sex). The small average difference between the groups is roughly equal to one year of accelerated aging at the level of the leukocyte, assuming an average yearly attrition of 31–66 bp [58], and given the measurement variance, this is not a meaningful difference. We had predicted that not all depressed subjects would be equally likely to show shortened telomeres, since the lifetime exposure to depression varied greatly between subjects, and since telomere length is believed to reflect cumulative history of cellular reproduction and of exposure to cytotoxic stimuli such as oxidation and inflammation and to stress. Therefore, as an *a priori* planned comparison, we examined telomere length within the depressed group as a function of cumulative lifetime duration of depression, corrected for age and sex. Next, as a secondary (exploratory) test of our hypothesis, we dichotomized lifetime exposure to depression into a categorical independent variable, comparing the control subjects (N = 17) to the depressed subjects in the highest half of lifetime depression duration (n = 9), while controlling for age and sex. Telomere length within the entire depressed group was significantly inversely correlated with lifetime depression duration (controlling for age and sex); individuals with greater lifetime duration of major depression had significantly shorter telomeres (F =  4.70, p<0.05) (Fig. 1). This relationship remained statistically significant when lifetime and current tobacco use and BMI were additionally controlled. When depressed individuals in the upper half of lifetime exposure to depression (≥9.2 years cumulative duration, n =  10) were compared to controls, significant differences in telomere length were observed (controls: 5141±282 bp vs. depressed: 4860±349 bp; difference =  281 bp; F =  2.87, p =  0.05, controlling for age and sex). This difference also remained significant when lifetime and current tobacco use and BMI were controlled. This difference in mean telomere length (around 280 bp) is equivalent to approximately 7 years of “accelerated aging” at the level of the leukocyte. The fact that this difference is significant after controlling for age suggests that greater lifetime duration of depression was not simply a proxy for older age, which is also associated with shorter telomeres.

In an exploratory analyses, we also assessed the relationship of telomere length to the duration of the current major depressive episode. The correlation was in the expected direction (with longer episode duration associated with shorter telomeres), but this missed statistical significance (r = −0.44, p<0.09, controlling for age and sex). We also examined the relationship of telomere length to HDRS ratings and found no significant correlation (r = −0.10, p<0.80, controlling for age and sex). We also examined the impact of antidepressant treatment on the relationship between lifetime duration of depression and telomere length. Lifetime exposure to *untreated* depression remained significantly inversely correlated with telomere length (F =  3.62, p<0.05), but lifetime exposure to depression *while receiving antidepressants* was not (F = 2.50, p =  0.11). The latter relationship must be interpreted cautiously, however, since the lengths of time subjects had remained depressed while receiving antidepressant medication spanned a relatively short range (range: 0 to 31.2 months).

### Relationship of Telomere Length to Age and Sex by Diagnosis

PBMC telomere length was significantly and inversely correlated with age (independently of sex) in the combined subject group (r = −0.36, p<0.05) and within the MDD group alone (r = −0.58, p =  0.01) but not within the control group alone (r = −0.07, p =  0.79). Male, as opposed to female, gender was associated with significantly longer PBMC telomere length (independently of age) in the combined subject group (t =  2.09, p<0.05) and within the control group alone (t =  2.27, p<0.05) but not within the MDD group alone (t =  1.06, p =  0.30).

### Oxidative Stress and Inflammation

There were no significant between-group differences in measures of oxidative stress (F2-isoprostanes/Vit. C ratio) (depressed: 0.014±0.015; controls: 0.010±0.010; F =  0.50, ns) or inflammation (IL-6) (depressed: 0.84±0.82 pg/ml; controls: 0.73±0.37 pg/ml; F =  0.01, ns). Further, there were no significant correlations between lifetime depression duration (controlling for age and sex) and either the oxidative stress ratio (r = −0.07, ns) or IL-6 (r =  −0.10, ns).

### Relationship of Telomere Length to Oxidative Stress and Inflammation

**Figure 2:**
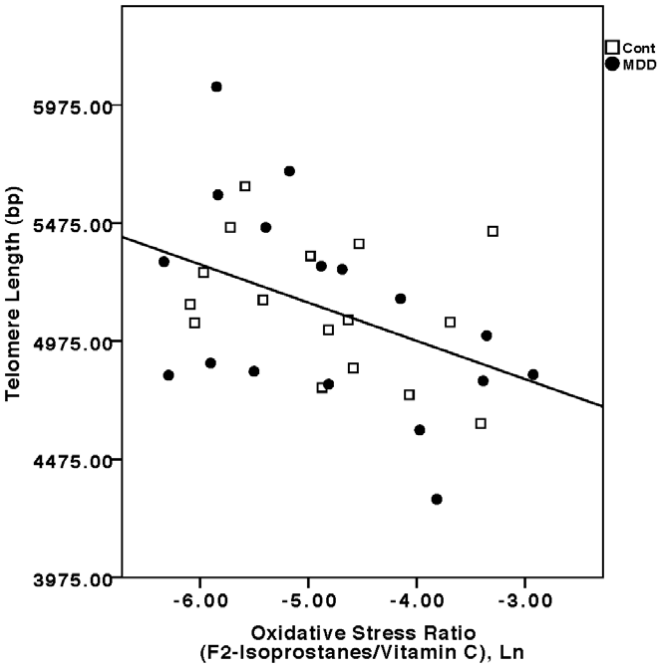
Relationship between the oxidative stress ratio (F-2 isoprostanes/Vitamin C concentrations, Ln transformed) and leukocyte telomere length (in base pairs, bp). Filled circles represent depressed subjects (“MDD”) (F =  6.04, p<0.01, controlling for age and sex), and open squares represent controls (“Cont”) (F =  4.38, p<0.05, controlling for age and sex). In the combined sample (depressed plus controls), the relationship was also statistically significant (F =  8.21, p<0.001, controlling for age and sex).

**Figure 3:**
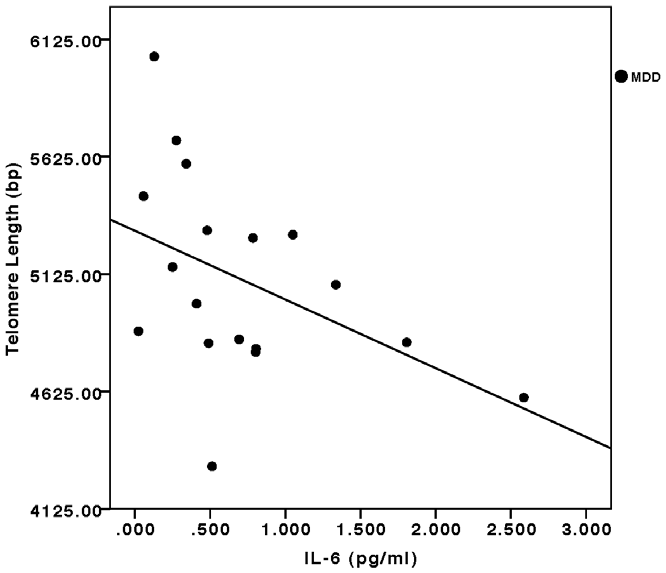
Relationship between serum IL-6 concentrations (pg/ml) and leukocyte telomere length (in base pairs, bp) in depressed subjects. (F =  3.29, p<0.05, controlling for age, sex and BMI). The relationship missed significance in the combined sample (depressed plus controls) (F =  2.45, p = 0.07, controlling for age, sex and BMI) and was not significant in the controls alone (not plotted) (F =  2.28, p =  0.13, controlling for age, sex and BMI).

In the combined sample (depressed plus control subjects), the oxidative stress ratio (F2-isoprostanes/Vitamin C) was significantly inversely correlated with telomere length (F =  8.21, p<0.001, controlling for age and sex) (Fig. 2). This relationship remained significant in the separate depressed (F =  6.04, p<0.01) and control groups (F =  4.38, p<0.05). Considering the components of this ratio separately, Vitamin C concentrations were significantly positively correlated with telomere length in the combined sample (F =  4.72, p<0.01) as well as in the individual depressed (F =  5.85, p<0.01) and control samples (F = 4.04, p<0.05) (all controlled for age and sex). F2-isoprostane concentrations were significantly negatively correlated with telomere length in the combined sample (F =  4.78, p<0.01, controlling for age and sex), but this relationship was only marginally significant in the separate depressed (F =  2.59, p<0.10) and control groups (F =  2.31, p =  0.13) due to low statistical power. IL-6 concentrations were significantly inversely correlated with telomere length in the depressed group (F =  3.29, p<0.05, controlling for age, sex and BMI) (Fig. 3), but not in the control group (F =  2.28, p =  0.13). In the combined sample, this relationship approached statistical significance (F =  2.45, p = 0.07, controlling for age, sex and BMI). After controlling for lifetime duration of depression, the relationships between the oxidative stress ratio and IL-6 concentrations and telomere length remained significant (F =  4.91, p<0.02, and F =  3.46, p<0.05, respectively).

## Discussion

Across a broad range of depressive chronicity, depressed individuals did not significantly differ from controls in leukocyte telomere lengths. However, individuals with more chronic courses of major depression had significantly shorter leukocyte telomeres than healthy controls, suggesting this may be a cumulative marker of chronic depression. Importantly, the relationship between telomere length and lifetime duration of depression was significant after age was controlled, indicating that longer exposure to depression was not simply a proxy for more advanced age, which is also associated with telomere shortening. Since telomere length has been proposed as a biomarker of cell aging and a predictor of health and longevity [38], [41], [42], [46], [47], this finding may underlie some of the excess medical morbidity and premature mortality seen in chronically depressed populations [1], [2], [3], [4], [5], [6], [7], [8], [9], [10], [11]. Depressed individuals with less chronic courses, however, showed no significant differences in telomere length, compared to controls. This argues against short telomeres representing a pre-existing risk factor for, or an invariant concomitant of, major depression. Rather, it suggests that telomere shortening may progress with longer exposure to depression. Prospective studies will be needed to explore this as well as the question of whether antidepressant treatment can attenuate this shortening.

Our finding that telomere shortening is directly related to the chronicity of depression exposure is similar to the finding of Epel et al. in caregivers [58]. Although there are clear differences between major depression and psychological stress, they, too, found that telomere shortening was directly correlated with the chronicity of caregiver stress, The study of affectively ill subjects by Simon et al. [55] found a significant shortening of telomere length in their whole affectively ill sample rather than in just a chronic subgroup, as we did. However, it is important to note that the average lifetime duration of affective illness in their depressed sample was 31.8 ±11.2 years, compared to the average lifetime duration of 13.0±11.2 years in our sample. Accordingly, the estimate of accelerated aging in our more chronically depressed individuals is in line with the data from that study. One other study of telomere length in MDD did not state the chronicity of its sample [60]. The only prior study that did examine leukocyte telomere length as a function of lifetime depression did not find a significant relationship [61], but those data may not be comparable to ours, since they did not exclude periods of time during which subjects were not depressed that occurred after the first onset of MDD, and since medical illnesses and various neuropsychiatric conditions were not excluded [61].

The degree of telomere shortening we observed in the more chronically depressed individuals (those above the median of chronicity) corresponds to approximately seven years of “accelerated cell aging.” This degree of “acceleration,” accords fairly well with that described in stressed maternal caregivers (9–17 years) [58], in combined spousal and offspring caregivers (4–8 years) [59], and in depressed/affectively ill individuals (6 to 10+ years) [55], [61], assuming an average annual rate of telomere bp attrition of 31–66 (although some estimates suggest annual attrition rates of only 19–25 bp [59], [61]). While shortened telomere length in chronic depression is consistent with findings in other chronically stressed and depressed populations [55], [58], [59], [61], [102], this measure is not specific to chronic stress or depression and thus is not useful as a specific diagnostic “biomarker.”

While the mediating biochemical factors responsible for telomere shortening in chronic depression cannot be adequately assessed in a cross-sectional study, our findings of significant inverse correlations between telomere length and oxidative and inflammatory stress in the depressed subjects raise the possibility that these biochemical stressors contribute to telomere shortening in chronic depression. However, the direction of causality is not fully clear, as cells with shortened telomeres hyper-secrete pro-inflammatory cytokines [28], [103]. We found that oxidative stress was inversely correlated with telomere length in the depressed subjects and the controls, consistent with the reported shortening effect of oxidation on telomeres [36], [37], [38], [57], [104]. IL-6 concentrations were inversely correlated with telomere length in the depressed subjects but not in the controls. The reasons for this difference are unknown. It may be related to our small sample sizes, or to differences in specific relationships between inflammatory and anti-inflammatory cytokines in depressed individuals vs. controls [71]. The lack of significant correlations between lifetime depression duration and both the oxidative stress ratio and IL-6 does not argue against the mechanistic hypothesis we put forward, since the hypothesis does not require that oxidative stress and inflammation be progressive across the life course of MDD. The lack of significant between-group differences in oxidation and inflammation, measured cross-sectionally, also does not argue against our mechanistic hypothesis. Although speculative, it is possible that depressed individuals are more sensitive to the telomere-shortening effects of oxidation and inflammation due to some other biochemical “co-factor,” or due to a lack of counter-regulatory anti-oxidant and anti-inflammatory activities. Regarding the first possibility, emerging studies are suggesting enhanced susceptibility of cells from depressed individuals to apoptosis [105]. Regarding the second possibility, we and others previously reported significantly low serum IL-10 levels in MDD [71], [106], along with an elevated IL-6/IL-10 ratio in MDD, despite no significant elevations in serum IL-6 levels [71]. IL-10 is an anti-inflammatory/immuno-regulatory cytokine, and data from that study suggested a perturbed balance between pro- and anti-inflammatory activity in MDD [71]. Similarly, studies have previously shown not only increased oxidative activity in MDD, but decreased counter-regulatory anti-oxidant activity [107], [108], [109], [110] in MDD.

Our findings, along with the literature reviewed below, suggest that chronic inflammation and oxidation may be mechanisms by which chronic depression can result in shortened telomeres. Significant increases in oxidative stress [62], [63], [65], [66], [67] and inflammatory stress [16], [17], [71], [72], [111], [112] have been described in many, but not all, studies of major depression. To the extent oxidative and inflammatory stress are chronically increased in depressed individuals, or to the extent depressed individuals are more sensitive to such cytotoxic stimuli or have a compromised ability to recover from oxidative or inflammatory damage, these stressors could contribute to telomere shortening [113], [114], [115], [116]. Conversely, leukocyte telomere shortening, resulting in immunosenescence and impaired leukocyte function, can lead to increased inflammatory cytokine output [28] and to increased oxidative stress [115], thus forming a vicious cycle [16]. The significant correlations we observed between oxidative and inflammatory stress and telomere length have not been previously reported in individuals with MDD, but they are consistent with relationships between oxidative and inflammatory stress and telomere length in other populations. It is possible that leukocyte telomere shortening occurs across conditions that are characterized by chronic exposure to oxidation and/or inflammation or by increased leukocyte turnover [49], [58], [59], [117], [118], [119], [120].

### Limitations

The major strengths of the present study are the use of physically healthy, well characterized, matched subjects, the prospective recruitment, the assessment of important covariates such as BMI, exercise and nicotine consumption, the exclusion of psychotropic and other medications that might have influenced our results, the assessment of depressive chronicity and the assessment of potential biochemical mediators. The major limitations are the small sample size, the use of self report to assess lifetime depression duration and the lack of longitudinal data. While the failure to discern an overall effect of depression (independent of chronicity) on telomere length may be due to the lesser chronicity of our sample compared to others [55], it is also possible that our small sample size lacked the statistical power to detect the effect. The effect size (Cohen's d) of telomere differences in the study by Simon et al. [55] was 0.74, in the study by Lung et al. [60] was 0.83, and in the study by Hartmann et al. [61] was 0.59 (effect sizes calculated from published data). The sample size used in the present study would have had between 40% –66% power to detect effects of these magnitudes. Due to our small sample size, especially the size of the sub-sample of depressed subjects with more chronic depressions, our results should be considered preliminary and in need of replication with a larger sample. There are several additional caveats in interpreting our data. First, the relationship between peripherally-measured inflammation and oxidation and such processes in the central nervous system is complex [121], [122], [123], [124], [125], [126], and we make no extrapolation of our results to CNS oxidation or inflammation. It should be noted, however, that telomere length is reduced in hippocampal CA1 neurons in Alzheimer's disease, and this has been postulated to result, at least in part, from oxidative stress [127]. Second, it is not known whether telomere length observed in leukocytes is related to telomere length in other cell types or tissues [128], including the central nervous system [129]. However, shortened telomeres in peripheral blood leukocytes were significantly correlated with smaller hippocampal volumes and with declining cognitive function among participants in the Nurses' Health Study [130]. The effects of depression on telomere lengths of specific leukocyte subpopulations and on specific tissues should be examined in future studies. Third, it remains to be determined if our observed telomere length findings represent changes on a per-cell basis or, rather, differences in the relative proportions of circulating blood cell types, since subpopulations of leukocytes (e.g., CD8+CD28- leukocytes) differ in average telomere lengths [131]. It will be important in future studies to determine which specific leukocyte subpopulations are mediating the observed effects. Lastly, since childhood adversity (e.g., childhood sexual abuse) is associated with shortened telomeres [132], [133], [134], and since childhood adversity is more common in depressed populations than in controls, such factors could have impacted our data, independent of the effects of depression.
